# Characterization of thermostable serine hydroxymethyltransferase for β-hydroxy amino acids synthesis

**DOI:** 10.1007/s00726-022-03205-w

**Published:** 2022-12-17

**Authors:** Ilma Fauziah Ma’ruf, Elvi Restiawaty, Syifa Fakhomah Syihab, Kohsuke Honda, Akhmaloka

**Affiliations:** 1 Doctoral Program of Chemistry, Faculty of Mathematics and Natural Sciences, Institut Teknologi Bandung, Bandung, Indonesia; 2 Biochemistry Research Group, Faculty of Mathematics and Natural Sciences, Institut Teknologi Bandung, Bandung, Indonesia; 3 Chemical Engineering Process Design and Development Research Group, Faculty of Industrial Technology, Institut Teknologi Bandung, Bandung, Indonesia; 4 Faculty of Sports and Health Education, Universitas Pendidikan Indonesia, Bandung, Indonesia; 5 International Center for Biotechnology, Osaka University, Suita, Japan; 6 Department of Chemistry, Faculty of Science and Computer, Universitas Pertamina, Jakarta, Indonesia

**Keywords:** Serine hydroxylmethyltransferase, Cloning, Characterization, Synthesis of β-hydroxy amino acids

## Abstract

β-hydroxy amino acids, such as serine, threonine, and phenylserine, are important compounds for medical purposes. To date, there has been only limited exploration of thermostable serine hydroxylmethyltransferase (SHMT) for the synthesis of these amino acids, despite the great potential that thermostable enzymes may offer for commercial use due to their high stability and catalytic efficiencies. *ITBSHMT_1* (ITB serine hydroxylmethyltransferase clone number 1) from thermophilic and methanol-tolerant bacteria *Pseudoxanthomonas taiwanensis* AL17 was successfully cloned. Biocomputational analysis revealed that ITBSHMT_1 contains Pyridoxal-3′-phosphate and tetrahydrofolatebinding residues. Structural comparisons show that ITBSHMT_1 has 5 additional residues VSRQG on loop near PLP-binding site as novel structural feature which distinguish this enzyme with other characterized SHMTs. In silico mutation revealed that the fragment might have very essential role in maintaining of PLP binding on structure of ITBSHMT_1. Recombinant protein was produced in *Escherichia coli* Rosetta 2(DE3) in soluble form and purified using NiNTA affinity chromatography. The purified protein demonstrated the best activity at 80 °C and pH 7.5 based on the retro aldol cleavage of phenylserine. Activity decreased significantly in the presence of 3 mM transition metal ions but increased in the presence of 30 mM β-mercaptoethanol. ITBSHMT_1 demonstrated Vmax, Km, Kcat, and Kcat/Km at 242 U/mg, 23.26 mM, 186/s, and 8/(mM.s), respectively. The aldol condensation reaction showed the enzyme’s best activity at 80 °C for serine, threonine, or phenylserine, with serine synthesis showing the highest specific activity. Biocomputational analysis revealed that high intramolecular interaction within the 3D structure of ITBSHMT_1 might be correlated with the enzyme’s high thermal stability. The above data suggest that ITBSHMT_1 is a potential and novel enzyme for the production of various β-hydroxy amino acids.

**Supplementary Information:**

The online version contains supplementary material available at 10.1007/s00726-022-03205-w.

## Introduction

Serine hydroxylmethyltransferase/SHMT (EC 2.1.2.1) is one of the most crucial enzyme one-carbon metabolic pathways (Giardina et al. 2018; Lakhssassi et al. 2020; Nogues et al.^2020^; Nguyen et al. 2021; Liu et al. 2022). This enzyme is ubiquitous, present in both of procaryotes and eucaryotes. Therefore, it is an important target for anticancer (Ubonprasert et al. 2019; Geeraerts et al. 2021; García-Cañaveras et al. 2021), antiplasmodial (Witschel et al. 2015) and antibacterial (Makino and Iwama 2022). SHMT belongs to the aspartate aminotransferase superfamily (Type I fold PLP-dependent enzymes) along with threonine aldolase and alanine racemase. Typically, a PLP-dependent enzyme frequently catalyzes a wide range of reactions, making it a potential biocatalyst for the production of a variety of compounds (Di Salvo et al. 2013). Among the aspartate aminotransferase superfamily, extensive study has been conducted into the exploration and engineering of threonine aldolase as a biocatalyst for β-hydroxy amino acids (Fesko 2016). SHMT shows a primary role in catalyzing the tetrahydrofolate (THF)-dependent conversion of serine to glycine. Additionally, it can catalyze the THF-independent threonine aldolase activity that interconverts β-hydroxy amino acids into glycine and aldehydes (Chiba et al. 2011; Angelaccio 2013; Ruszkowski et al. 2018; Nonaka et al. 2019; Ma’ruf et al. 2021). Several SHMTs from microbes, such as *Hydrogenobacter thermophilus* TK‐6 (Chiba et al. 2011), *Escherichia coli* K12 (Zhao et al. 2011), and *Psychromonas ingrahamii* (Angelaccio et al. 2014), have been reported to show retro aldol cleavage activity toward various β-hydroxy amino acids. On threonine aldolase, the reaction equilibrium was successfully shifted into β-hydroxy amino acids synthesis by adding glycine in excessive amounts (Fesko 2016). Owing to the similarity of its catalytic activity with that of threonine aldolase, SHMT also has considerable potential to catalyze the synthesis of β-hydroxy amino acids and their derivatives.

β-hydroxy amino acids consist of canonical and non-canonical amino acids that have direct biological activity or function as a building block for various medical compounds (Di Salvo et al. 2013). For example, l-serine has been used in the treatment of a neurometabolic disease, namely serine deficiency disorder (Méneret et al., 2015). l-threonine is found in polypeptide drugs, such as exenatide (for the treatment of type 2 diabetes mellitus), enfuvirtide (inhibitor of HIV fusion), sermorelin (for the treatment of dwarfism), and ceruletide (stimulator of digestive secretions and smooth muscle) (Szcześniak et al., 2014). β-phenylserine is a precursor of the l-Threo-3,4-dihydroxyphenylserine (DOPS) drug used to treat Parkinson’s disease (Lee et al., 1994).

Amino acids are usually produced through protein hydrolysis, chemical synthesis, or biotechnological approaches, such as fermentation and the use of microbial enzymes. Among these, biotechnological approaches have been extensively employed for the bulk synthesis of amino acids (Ivanov et al. 2013). Currently, the synthesis of proteinogenic β-hydroxy amino acids depends on a fermentation process that uses *Corynebacterium glutamicum* and *E. coli* to produce L-threonine (Anushree and Nampoothiri 2015) and L-serine (Wang et al. 2020; Zhang et al. 2020). However, none of the processes uses thermophilic microorganisms or thermostable enzymes.

The discovery of thermophilic microorganisms and extremozymes had a huge impact on the field of biochemical engineering. Thermostable enzymes are preferable for use in the bioprocess industry since at high temperatures, the process may increase the conversion rate, substrate solubility, and reduce contamination of mesophilic organisms (Restiawaty et al. 2011; Balan et al. 2012). In addition, thermostable enzymes are commonly resistant to organic solvents, proteases, chaotropic agents, and detergents (Angelaccio 2013). Several studies have reported extensive exploration of the thermophilic microorganisms and their enzyme application through cultivation or metagenomic cloning methods (Widhiastuty et al. 2009; Nurhasanah et al. 2015; Suharti et al. 2014, 2021). Certain thermophilic microorganisms were isolated from the thermogenic phase of the composting process and showed methanol tolerance (Syihab et al. 2015). One of the isolates (AL17) was characterized as close to *Pseudoxanthomonas taiwanensis.*

In this study, AL17 isolate was used as a source of the SHMT gene since the bacteria have been shown to have thermophilic and methanol tolerance properties. Various findings have shown that SHMT from methylotroph is able to catalyze in vivo or in vitro serine production (Izumi et al. 1993; Hagishita et al. 1996; Jiang et al. 2013a, 2013b). Serine was intra-cellularly produced from methanol and glycine, employing the serine pathway, which includes two key enzymes (SHMT and methanol dehydrogenase) (Izumi et al. 1993; Hagishita et al. 1996). For the in vitro process, SHMT was heterologously expressed to convert glycine and formaldehyde into serine in the presence of THF (Jiang et al. 2013a, 2013b). In this paper, we reported that ITBSHMT_1 was able to synthesize various β-hydroxy amino acids such as serine, threonine and phenylserine.

## Materials and methods

### Isolation of bacterial genomic DNA

Bacteria (AL17) were grown overnight in MC broth (0.5% beef extract, 0.5% yeast extract, 0.1% CaCl_2_.2H_2_O, and 0.1% NaCl) and shaken at 150 rpm at 55 °C. The culture was reinoculated into a new MC broth (18 h 150 rpm, 55 °C). After that, the cell was separated by centrifugation at 6000 x*g* for 5 min. Genomic DNA was extracted from 20 to 30 mg of cell pellet using a previous method (Syihab et al. 2015). 

### Primer design and polymerase chain reaction

To isolate the SHMT gene, a pair of primers, namely PXSHMT_F (5′-ATGTACTCGCGTGATGCCCG-3′) and PXSHMT_R (5′-GCTCAGCCGTAGACCGG-3′), was constructed based on the SHMT gene from the whole genome sequence data of *P. suwonensis* 11–1 (accession number: CP002446.1). The polymerase chain reaction (PCR) was initiated with a 5-min initial denaturation at 95 °C, followed by 28 reaction cycles (95 °C for 30 s, 58 °C for 30 s, 72 °C for 1 min); the final step was a 10-min elongation process at 72 °C.

### Cloning of *ITBSHMT_1*

The amplicon was ligated into pJET 1.2/blunt plasmid (Thermo Fisher Scientific). The plasmid solution was then used to transform *E. coli* TOP 10. Positive transformants were selected in LB medium (100 ug/mL ampicillin). A single colony was then inoculated into a new LB medium in a Petri dish. The bacterial colony was resuspended in rapid disruption buffer (10 mM NaOH, 5 mM EDTA, 0.05% Bromophenol Blue, 0.25% SDS, 10% sucrose, and 60 mM KCl) and boiled for 5 min. The DNA solution was analyzed using agarose gel electrophoresis. The positive recombinant plasmid was isolated and digested using *Bgl*II to identify the presence of the inserted gene. Sequencing services provided by First Base (Malaysia) were used to establish the nucleotide sequence. Prior to the insertion of *ITBSHMT_1* into expression vector pET30a (+) at *Nde*I and *Xho*I restriction sites, a pair of primers, namely PXSHMT_FEX (5′-CAACATATGTACTCGCGTGATGCC-3′) and PXSHMT_REX (5′-AGACTCGAGGCCGTAGACCGGGTAC-3′), was used to perform the sub-cloning process. After double digestion with the two restriction enzymes, ligation was catalyzed by T4 DNA ligase (Thermo Scientific). The result was then transformed into *E. coli* Rosetta 2 (DE3).

### Expression and purification of protein

*E. coli *Rosetta 2(DE3) harboring pET-*ITBSHMT_1* was grown on LB broth (50 µg/mL kanamycin and 30 µg/mL chloramphenicol) overnight (150 rpm). The bacterial suspension was then reinoculated into a new LB medium up to OD_600_ 0.6. Recombinant protein expression was induced using 1 mM IPTG (25 °C, 150 rpm, 20 h) and protein was isolated using the lysozyme-heating method according to the following procedure. Cell pellet (100 mg) was resuspended in 1 mL of 50 mM phosphate buffer pH 7.5 (250 mM NaCl, 1 mg/mL lysozyme) and shaken at 150 rpm 25 °C, 2 h. This was followed by heating at 70 °C for 10 min and then a centrifugation process (12,000 x*g*, 4 °C, 5 min). The purification was undertaken based on a previous method (Suharti et al. 2021; Ma’ruf et al. 2021), while the dialysis process was carried out to discard imidazole from the enzyme solution.

### Electrophoresis and zymography

SDS-PAGE analysis was performed according to a previous method (Suharti et al. 2021). For native PAGE and zymography analysis, the electrophoresis gels were treated without the addition of SDS and the boiling step (Syihab et al. 2015).

Proteins on native PAGE were stained based on a previous method (Ulevitch and Kallen 1977) with slight modifications — we used Coomassie brilliant blue/CBB instead of aniline blue black. One gel was stained with standard CBB while another was stained based on SHMT activity (zymography). Substrate solution (50 mM sodium phosphate buffer pH 7.5, 25 mM sodium sulfate, 50 mM DL-phenylserine, and 50 μM PLP) was added to native-PAGE gel and incubated for 20 min at 60 °C. Disposal of the substrate solution was followed by staining with 0.2% dinitrophenylhydrazine solution (dissolved in 2 M HCl) for 5 min at 25 °C.

### Enzyme activity assay

The enzyme activity assay was conducted based on a previous method (Jiang et al. 2013a) with certain modifications. Different from the previous method, we used TCA 1% to terminate the enzymatic reaction while the measurement was performed at *λ* 292 nm instead of *λ* 279 nm. The complete method is described as follows: 1 mL of the reaction mixture (2 μL of the enzyme, 50 mM phosphate buffer pH 7.5, 25 mM sodium sulfate 50 mM DL-phenylserine, and 50 μM PLP) was incubated for 5 min at 70 °C, then trichloroacetic acid/TCA (final concentration 1%) was added to terminate the reaction. Benzaldehyde (reaction product) was monitored at *λ* 292 nm. One unit (U) of enzyme activity was defined as the amount of enzyme that catalyzed the release of 1 µmol of benzaldehyde per minute.

The enzyme activity at various pH was determined using two buffers: sodium phosphate (pH 5.5–8.5) and phosphate–NaOH (pH 9.5–11.5) at 70 °C. The enzyme activity at various temperatures (30–100 °C) was observed at pH 7.5. 3 mM of metal ions and chemical reagents were added to the enzymatic mixture to observe the effect of each chemical on ITBSHMT_1 activity (pH 7.5, 80 °C).

The effect of various substrate concentrations (1.25–90 mM DL-phenylserine) was determined at pH 7.5, 80 °C. The kinetic parameters were predicted via non-linear regression of the resulting Michaelis–Menten plot using R software (https://www.R-project.org/).

### Serine, threonine, and phenylserine synthesis

The initial velocity of serine, threonine, or phenylserine synthesis was measured in a 500 µL reaction with the following buffer solution components: 5.54 U SHMT, 50 mM phosphate buffer pH 7.5, 25 mM sodium sulfate, 50 mM glycine, 2 mM THF (for serine synthesis only), 50 mM formaldehyde (for serine synthesis), 10 mM acetaldehyde (for threonine synthesis), 1 M benzaldehyde (for phenylserine synthesis), 100 mM β-mercaptoethanol, and 0.3 µM PLP. The mixture was incubated at various temperatures (50–100 °C) for 5 min before the enzymatic reaction was terminated with the addition of 1% TCA.

### HPLC assay

Amino acidphenylisothiocyanate/PITC derivatization was performed based on a previous method (Kameya et al. 2007; Ma’ruf et al. 2021) with certain modifications; for example, we used a dry bath instead of a vacuum concentrator. 10 µL of reaction product was added to 10 µL of 50 mM L-alanine in a microtube and then dried at 80 °C in a dry bath for 30 min. The sample was dissolved with 20 µL of ethanol: milliQ: triethylamine (2: 2: 1) and dried for 20 min at 80 °C. The precipitate was then dissolved with 20 µL of ethanol: milliQ: triethylamine: PITC (7: 1: 1: 1). This was followed by incubation at room temperature for 20 min and a drying process for 25 min at 80 °C. The sample was dissolved with 500 µL of methanol. HPLC analysis was performed by Agilent Technologies 1260 Infinity HPLC system using ZORBAX Eclipse XDB-C18 column (4.6 × 250 mm, 5 µm). Two types of buffers–A buffer (15 mM phosphate buffer pH 7) and B buffer (methanol LC grade)–were used for the elution process. The samples were run for 36 min, with B buffer gradient from 0 to 75% for the first 30 min. The buffer concentration was then reduced to 0% for the remaining 6 min.

### Computational analysis

BioEdit software (Hall 1999) was used to conduct multiple sequence alignment. MEGA-X software with the Neighbor-Joining method (Kumar et al. 2018a, b) was used to build a phylogenetic tree. Secondary structure alignment was performed using Promals3D website (http://prodata.swmed.edu/promals3d/). The 3D model of ITBSHMT_1 was generated using the SWISS-MODEL website (Waterhouse et al. 2018). Protein minimization, protein visualization and in silico mutation were performed using UCSF Chimera software (Pettersen et al. 2004). Pyridoxal phosphate Glycine (PLG), 5-formyl-tetrahydrofolate/5-formyl-tetrahydropteroylglutamate (FFO) and 5-formyl-tetrahydropteroyltriglutamate (TGF) were downloaded from the PDB website (https://www.rcsb.org/). Pyridoxal Phosphate Phenylserine (PLF) was built, and energy was minimized using Marvin Chem software (https://chemaxon.com/products/marvin). Docking was performed using Autodock Vina software (Eberhardt et al. 2021) and visualized using Ligplot plus software (Laskowski and Swindells 2011). Hydrophobic clusters, salt bridges, and hydrogen bonds on the protein structure were identified using the ProteinTools server (https://proteintools.uni-bayreuth.de). The transition metal-binding sites of the enzyme were analyzed using MIB: Metal Ion-Binding site prediction and docking server (http://bioinfo.cmu.edu.tw/MIB/).

### Statistical analysis

At least three independent experiments were performed to obtain mean and standard deviation of data. Statistical analysis has been conducted using *T* Test and one-way ANOVA. *P* value ≤ 0.05 is considered significant.

## Results

### Clone and homology of ITBSHMT_1

**Fig. 1:**
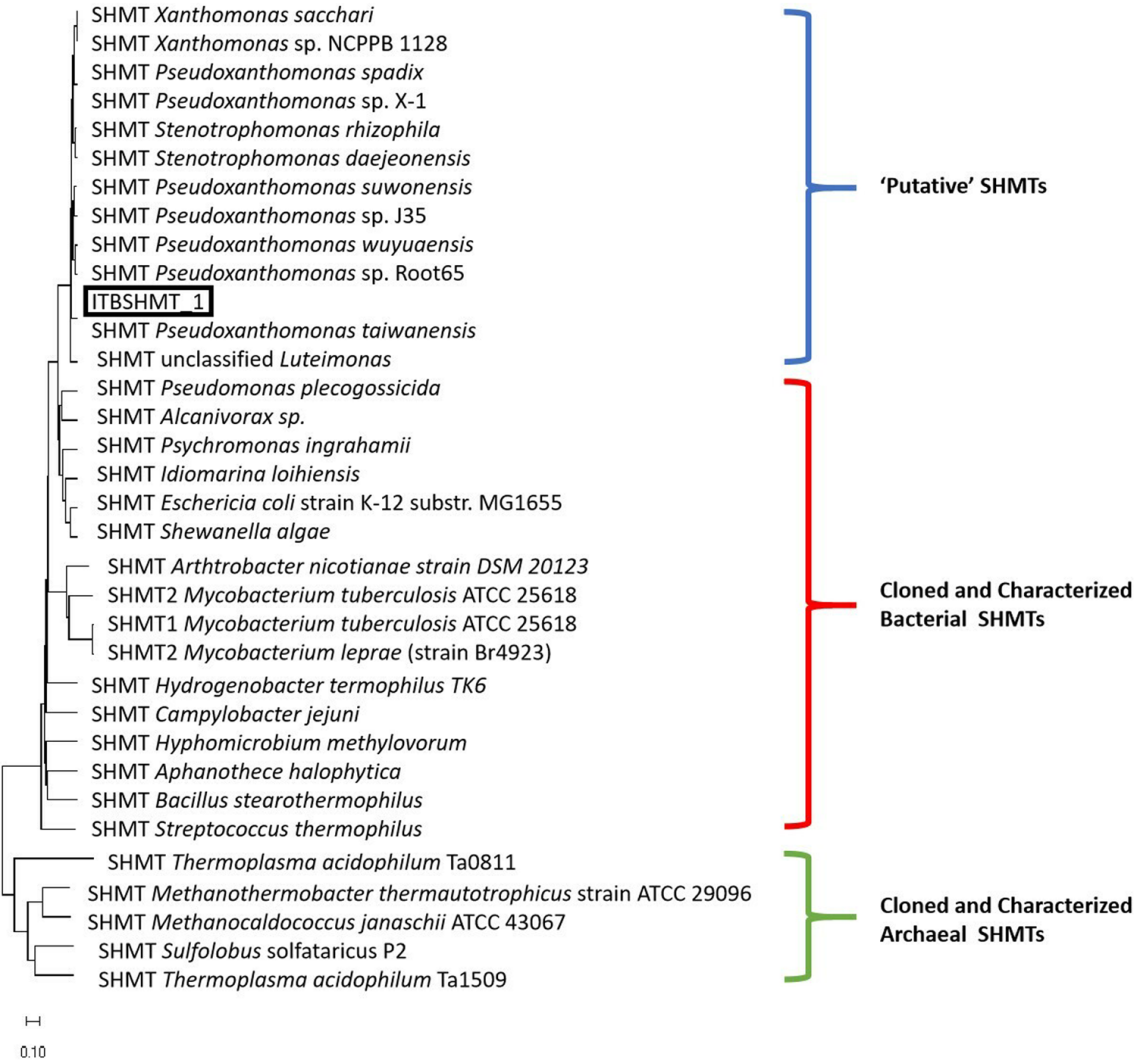
Phylogenetic Tree constructed by MEGA X Program using Neighbor-Joining method (Kumar et al. 2018b). ITBSHMT_1 (black square), ‘putative’ SHMTs (blue bracket), clone and characterized bacterial SHMTs (red bracket) and clone and characterized archaeal SHMTs (green bracket) were indicated

The SHMT gene from local isolate AL17, namely *ITBSHMT_1*, was cloned by PCR amplification and sequenced. An open reading frame appeared with 1,269 base pairs encoding 422 amino acids. Homological analysis using the BLAST program toward NCBI data (https://blast.ncbi.nlm.nih.gov/Blast.cgi) revealed that the protein demonstrated 99.05% identity with SHMT of *P. taiwanensis* (accession number: WP_162123706.1). The nucleotide sequence of *ITBSHMT_1* was deposited into the gene bank with accession number MN688201.1. A phylogenetic tree was generated using the MEGA-X program (Kumar et al. 2018b) and showed that the archaeal, bacterial, and ‘putative’ bacterial SHMT were distinctly grouped. Moreover, *P. taiwanensis* SHMT and ITBSHMT_1 were located in different cluster among the other ‘putative’ SHMT (Fig. 1).

Homological analysis based on alignment with other known SHMTs revealed that ITBSHMT_1 contains a PLP and tetrahydrofolate (THF)-binding site (Supplemental Fig S1). A 3D structure model of ITBSHMT_1 was constructed based on the crystal structure of *E. coli* SHMT (1dfo). The result showed that ITBSHMT_1 is a dimer protein with PLP- and THF-binding sites facing in opposite directions (Supplemental Fig S2).

In further analysis to prove the predicted PLP- and THF-binding sites, the 3D structure model was docked with pyridoxal phosphate glycine (PLG/PLP analog) and 5-formyl-tetrahydrofolate (FFO/THF analog). The residues involved in PLP- and THF-binding were visualized using the Ligplot plus program (Laskowski and Swindells 2011). The result showed that the residues for PLP binding, such as G98, S99, D200, H203, G268, and R368, formed hydrogen interaction with the ligand (PLG) (Supplemental Fig S3A). Docking with FFO to the protein structure also led to the formation of a hydrogen bond between the ligand and predicted residues of the THF-binding site, such as L121, G125, L127, and N352 (Supplemental Fig S3B). In addition, docking of 5-formyl-tetrahydropteroyltriglutamate (TGF) on FFO-binding site shows that there are residues, such as S35, A122, H126, L127, R132, N352 and R359 forming hydrogen bond with the polyglutamylated folate (Supplemental Fig S3C).

### Expression and purification of ITBSHMT_1

**Fig. 2:**
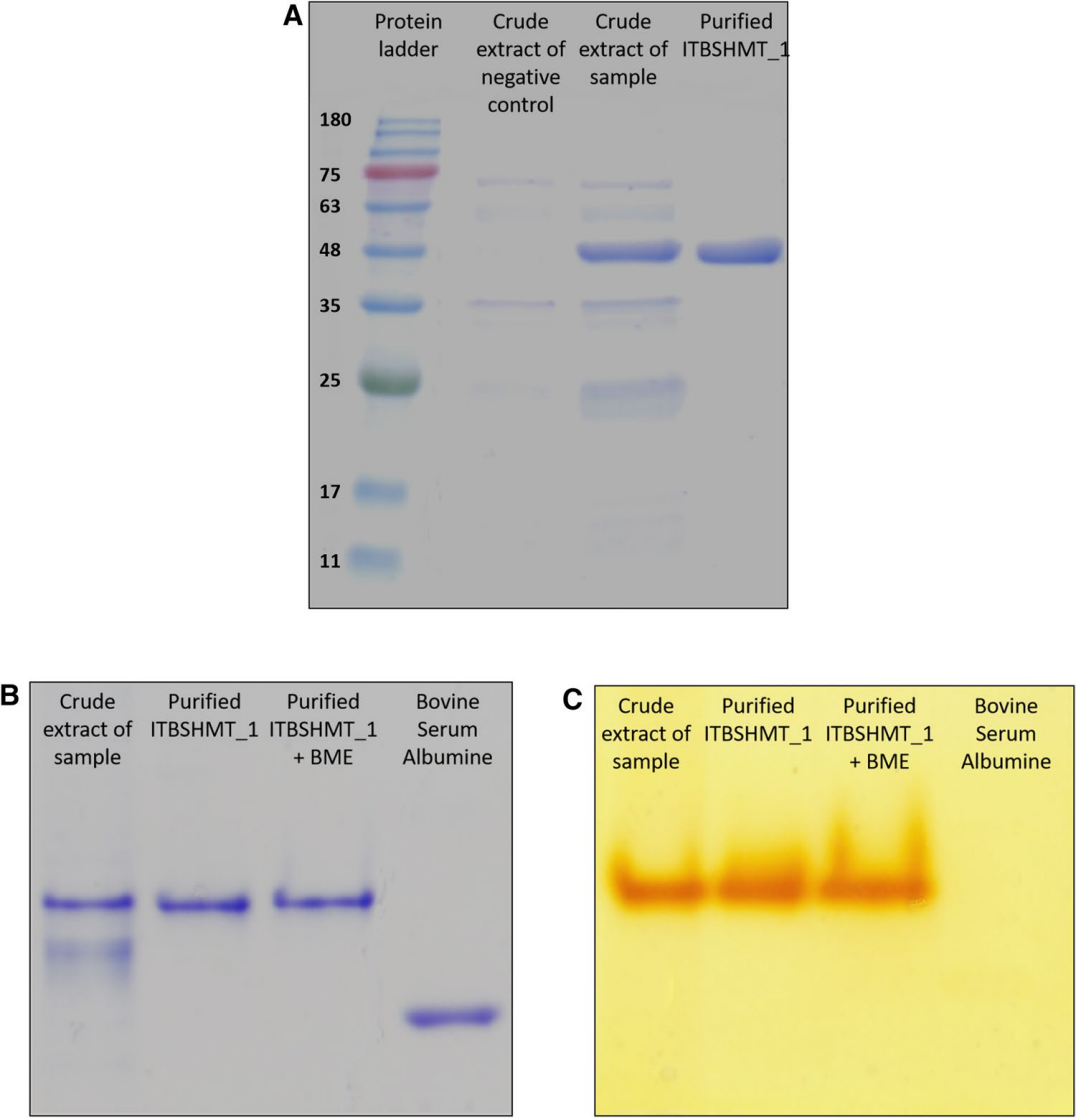
Electropherogram of crude extract and purified ITBSHMT_1. **A**. SDS-PAGE electropherogram, Coomassie brilliant blue staining **B**. Native-PAGE electropherogram, Coomassie brilliant blue staining, **C**. Native-PAGE electropherogram, zymography staining

*E. coli* Rosetta 2 (DE3) harboring pET-ITBSHMT_1 was grown and induced using IPTG. Lysozyme was used to lyse the cell (Yohandini et al. 2008) followed by a 10 min heating process at 70 °C. Centrifugation was then conducted to separate the supernatant containing the enzyme and cell debris. The method produced crude extract with a background containing few proteins as the heating process led to denaturation and precipitation of the heat-labile protein (Neddersen and Elleuche 2015). Subsequently, the ITBSHMT_1 protein was successfully purified using the NiNTA affinity chromatography method (Fig. 2A). A single band appeared on the SDS-PAGE electropherogram with molecular weight/MW ~ 47 kDa. Qualitative analysis demonstrated that the crude extract and purified protein continued to show activity to cleave, DL-phenylserine into glycine and benzaldehyde on zymographic assay on native PAGE, and benzaldehyde was reacted with 2,4-dinitrophenylhydrazine to form a yellow precipitate (Fig. 2C). Comparison with Bovine Serum Albumin/BSA (MW: ~ 66.5 kDa) revealed that the native ITBSHMT_1 had a higher MW than BSA (Fig. 2B). This result supports previous analysis (Supplemental Fig S2) that native ITBSHMT_1 is a dimer protein with MW of approximately 94 kDa. On the other hand, quantitative analysis revealed that the specific activity of the purified protein was almost four times higher compared to that of the crude extract (Supplemental Table S1).

### Activity of ITBSHMT_1 on various pH and temperatures and measurement of the enzyme’s kinetic properties

**Fig. 3:**
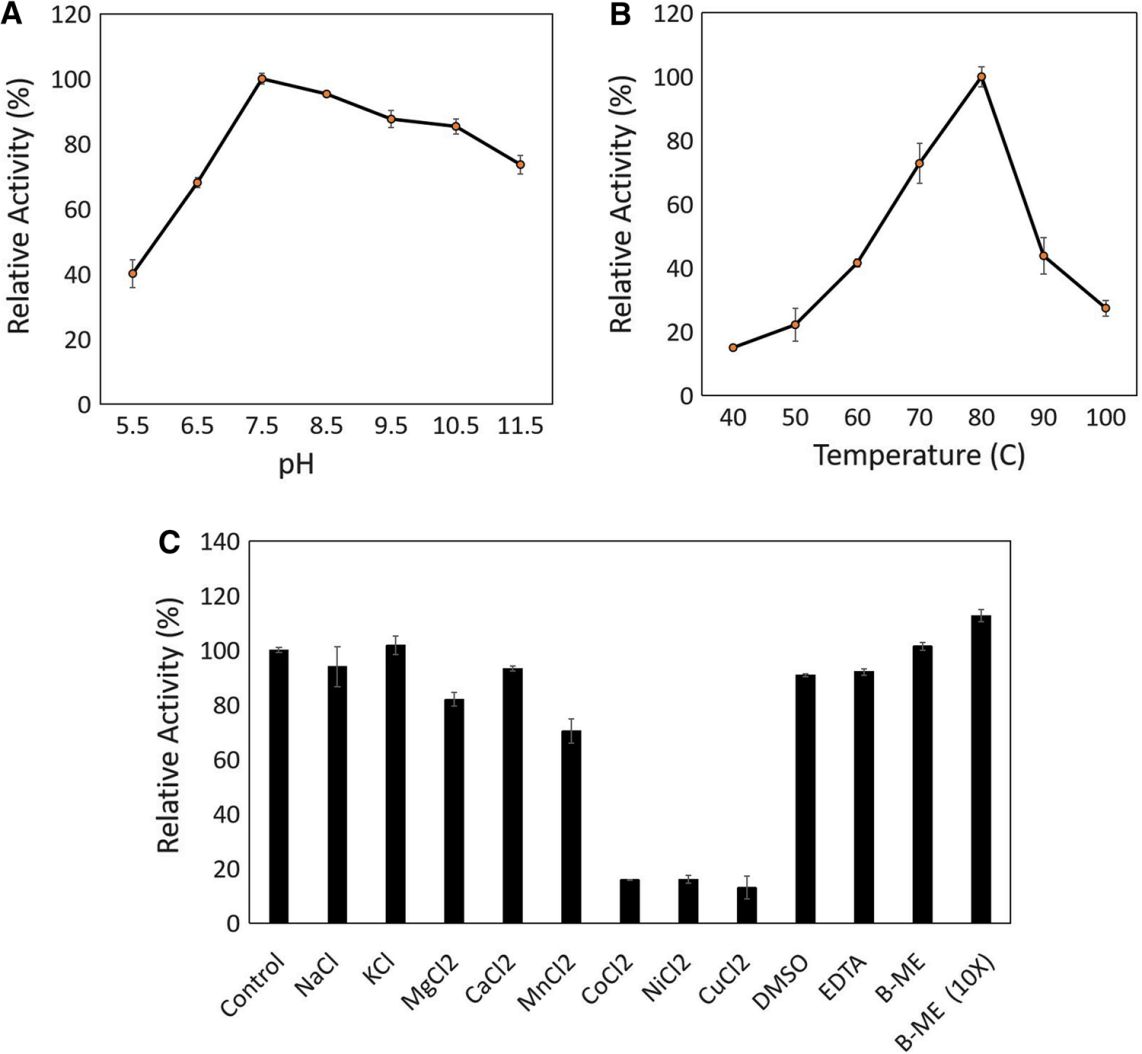
**A**. Relative activity of ITBSHMT_1 on variation of pH (70 °C, retroaldol cleavage of DL-phenylserine). **B**. Relative activity of ITBSHMT_1 on variation of temperature (pH 7.5, retroaldol cleavage of DL-phenylserine). **C**, Relative activity of ITBSHMT_1 in the presence of metal ions and chemical reagents (80 °C, pH 7.5, retroaldol cleavage of DL-phenylserine). All of experiment considered as significant (P value ≤ 0.05) except for addition of Na^+^, K^+^ and BME

A DL-phenylserine retroaldol cleavage reaction was conducted to characterize the enzyme. To probe the effect of pH on ITBSHMT_1 activity, an assay was conducted on a range of pH values from 5.5 to 11.5. ITBSHMT_1 exhibited optimum activity at pH 7.5 (Fig. 3A). There was a dramatic fall of up to 60% in enzyme activity at pH 5.5; however, up to 80% of activity was retained at pH 10.5. This suggested that in terms of activity, the enzyme has greater tolerance to alkaline conditions. Furthermore, 80 °C was the optimal temperature for ITBSHMT_1. At temperatures of 90 and 100 °C, the enzyme activity fell dramatically to 55 and 70% (Fig. 3B), respectively. After unveiling the thermal stability properties of ITBSHMT_1, the ProteinTools server (https://proteintools.uni-bayreuth.de) was used to analyze the intramolecular interaction within the 3D structure of the protein and the mesophilic SHMT from *E. coli* (1dfo). The result showed that ITBSHMT_1 has more hydrogen bonds, salt bridges, and hydrophobic clusters than *E. coli* SHMT (Table 2).

Further characterization of the kinetic parameters of ITBSHMT_1 showed that the enzyme exhibited Vmax (242 U/mg), Km (23.26 mM), Kcat (186 /s), and Kcat/Km (8 mM/s).

### In silico mutation of ITBSHMT and structural analysis

In silico mutation was conducted to predict effect of key binding residues mutation on protein structural stability (addition or reduction of intramolecular interactions) and cofactors-binding affinity on ITBSHMT_1. In silico mutation of conserve Tyrosin residue (YY) that has role as FFO- and PLG-binding site and deletion of unique fragment of ITBSHMT_1 (VSRQG) is predicted to change intramolecular interaction and or cofactors’s binding affinity (Table 2).

### Effect of metal ions, EDTA, and β-mercaptoethanol on activity of ITBSHMT_1

ITBSHMT_1 was assayed on a variation of metal ions and chemical reagents (Fig. 3C). The result demonstrated that transition metal ions (Co^2+^, Ni^2+^, Cu^2+^) led to a significant decrease in activity. Cu^2+^ led to the highest decrease in protein activity and was confirmed using biocomputational analysis. The Cu^2+^ binding site was analyzed using the Metal Ion-Binding server (http://bioinfo.cmu.edu.tw/MIB/) while the substrate (pyridoxal-5′-phosphate phenyl-serine/PLF)-binding site was analyzed using Autodock Vina software (Supplemental Fig S4). The result showed that there are similar residues between the Cu^2+^ and PLF-binding sites (Table 3). By contrast, alkaline metals (Na^+^, K^+^) did not appear to significantly affect the activity. In addition, 30 mM β-mercaptoethanol elevated enzyme activity was observed at around 12%.

### Synthesis of β-hydroxy amino acids

**Fig. 4:**
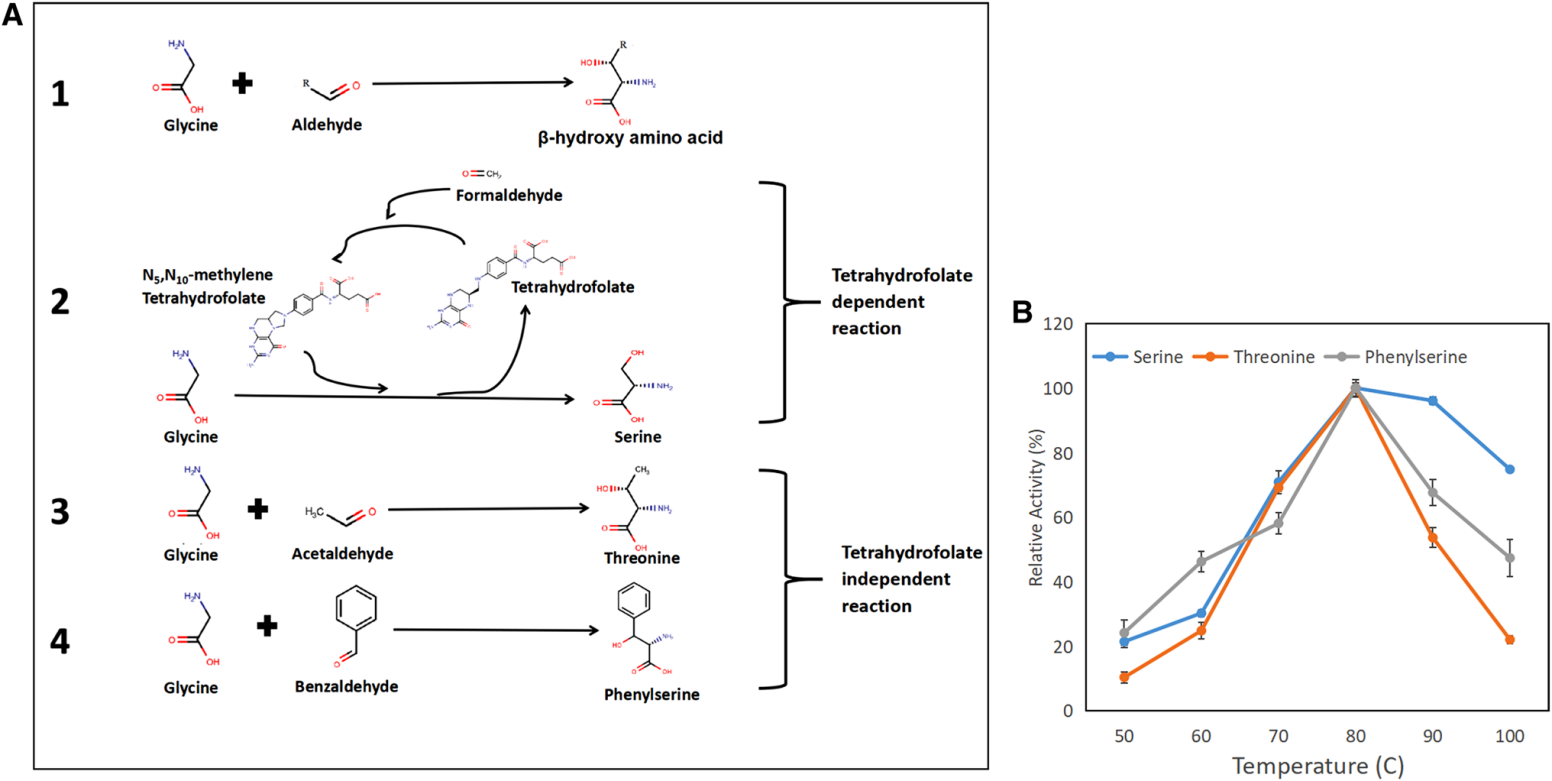
**A**. Mechanism of β-hydroxy amino acids synthesis by THF-dependent (2) and THF-independent reaction (3, 4). **B**. Relative activity of ITBSHMT_1 in serine, threonine and phenylserine synthesis on variation of temperature (150 rpm, pH 7.5)

It was found that SHMT catalyzed the THF-dependent and -independent reactions (Fig. 4A). Table 4 proves that serine synthesis did not occur in the absence of THF. Surprisingly, the addition of a reducing agent led to an almost 15-fold increase in the specific activity of serine synthesis. However, the synthesis of threonine and phenylserine did not appear to be significantly affected by the addition of THF. Moreover, the addition of a reducing agent increased the specific activity of threonine and phenylserine synthesis by almost 4 and 5 times respectively. ITBSHMT_1 activity to produce β-hydroxy amino acids (serine, threonine, and phenylserine) was observed at various temperatures, and the optimum enzyme activity was exhibited at 80 °C (Fig. 4B).

## Discussion

The phylogenetic tree (Fig. 1) suggests that the amino acid sequence of ITBSHMT_1 differs from both the ‘putative’ and characterized SHMTs. In addition, since no exploration of SHMT from *Pseudoxanthomonas* sp., further characterization of ITBSHMT_1 is needed. PLP-binding sites were conserved in all characterized SHMTs, while there was variation in the THF-binding residues, especially on the SHMT from archaea (Supplemental Fig S1) since archaea SHMT was notable in its use of modified folate instead of tetrahydrofolate as a cofactor (Angelaccio et al. 2014; Fesko 2016). Alignment with other known SHMTs demonstrated that ITBSHMT_1 contained residues for PLP binding at S35, Y55, Y65, Y70, G98, S99, H126, S175, D200, H203, T226, H228, K229, G268, and R368. Meanwhile, THF-binding residues were observed at E57, Y64, L121, G125, L127, and N353 (Supplemental Fig S1). The PLP- and THF-binding residues were similar to those on the crystalized structure of *E. coli* SHMT/1dfo (Scarsdale et al. 2000). In addition, the docking analysis (Supplemental Fig S3A and S3B) supported that ITBSHMT_1 uses PLP and THF as a cofactor. Docking analysis was also performed using 5-formyl-tetrahydro pteroyltriglutamate since the folate cofactor required poly-glutamylated tail to perform physiological activity (Fu et al. 2003) and the poly-glutamylated cofactor is predominant folate in all organisms (Guiducci et al. 2019). The result shows that pterin and p-amino benzoate moiety of this molecule is similar to those of binding site of FFO except for poly-glutamate tail-binding site (Supplemental Fig S3C).

The 3D structure model of ITBSHMT_1 (Supplemental Fig S2) supported the findings of previous studies that most procaryotic SHMT shows as an active dimer, while eucaryotic SHMT is active as a tetramer (Trivedi et al. 2002; Chaturvedi and Bhakuni 2003; Angelaccio 2013; Giardina et al 2015; Ruszkowski et al. 2018). Moreover, native PAGE analysis (Fig. 2B and Fig. 2C) also showed that the active form of ITBSHMT_1 was a dimeric protein. This phenomenon occurs because the bacterial SHMTs have two catalytic pockets on their dimer structure and the catalytic residues on each subunit A partially consist of residues from both subunits A and B, and vice versa (Scarsdale et al. 2000). The catalytic residues on each subunit of ITBSHMT_1 are described in Supplemental Table S2.

The characterization of ITBSHMT_1 demonstrated that the enzyme exhibits optimal activity at pH 7.5 and 80 °C. Comparing the data with previous findings revealed that most of the characterized SHMT showed an optimum pH in the range of neutral and moderate alkaline pH (pH 7–9) (Angelaccio et al. 2003, 2012; Zuo et al. 2007; Chiba et al. 2011; Waditee-Sirisattha et al. 2012; Jiang et al. 2013a; Jiang et al. 2013a; Yuan et al. 2014; Huang et al. 2015; Kumar et al. 2018a, b; Ma’ruf et al. 2021). SHMT is an intracellular enzyme, it is thus not surprising that ITBSHMT_1 shows optimum activity at pH 7.5 as intracellular enzymes usually have an optimum pH in the range of 7–8.0 (Booth 1985). In addition, to uncover the thermostable property of the enzyme, the ITBSHMT_1 3D protein model was compared with the established structure of mesophilic SHMT from *E. coli* (PDB id: 1dfo). The structural comparison revealed that ITBSHMT_1 has a higher number of hydrogen bonds, salt bridges, and hydrophobic clusters than 1dfo (Table 2). Previous findings have shown that an increase in hydrogen bonds, salt bridges, and hydrophobicity usually enhances protein thermal stability (Kumar et al. 2000; Rathi et al. 2014). Higher intramolecular interactions within ITBSHMT_1 than in the mesophilic counterpart might contribute to the protein’s high optimal temperature. Moreover, research into the kinetic parameters revealed that ITBSHMT_1 has approximately 42% catalytic efficiency compared to hyperthermostable SHMT from *H. thermophilus* TK-6, along with 54 and 9 times higher catalytic efficiency compared to mesophilic (*E. coli*) and psychrophilic (*P. ingrahamii*) SHMT (Table 1). This result demonstrates the potential application of ITBSHMT as a biocatalyst for industrial purposes since thermal stability and catalytic efficiency are the two most important characteristics of an enzyme (Yang et al 2018; Li et al 2021).

**Table 1:** Kinetic parameters of *ht*SHMT (Chiba et al. 2011), *mj*SHMT (Angelaccio et al., 2003), *e*SHMT (Contestabile et al., 2002), *pi*SHMT (Angelaccio et al, 2012), *il*SHMT (Kumar et al, 2018a, b) and ITBSHMT_1

	*Hydrogenobacter thermophiles* TK-6 SHMT (Chiba et al. 2011)	*Methanocaldococcus janaschii* SHMT (Angelaccio et al. 2003)	*Escherichia coli* SHMT (Contestabile et al., 2002)	*Psychromonas ingrahamii* SHMT (Angelaccio et al 2012)	*Idiomarina loihiensis* SHMT (Kumar et al 2018a, b)	ITBSHMT_1 (this study)
Substrate	dl-threo-phenylserine	dl-threo-phenylserine	dl-threo-phenylserine	l-threo-phenylserine	dl-phenylserine	dl-phenylserine
Condition	75 °C, pH 7.5	60 °C, pH 7.2	30 °C, pH 7.0	30 °C, pH 7.2	50 °C,pH 7.5	80 °C, pH 7.5
Kcat(/s)	93.8	59.6	2.78	14.2	1.51	186.18
Km (mM)	4.98	95	19	17.2	0.27	23.26
Kcat/Km (/s,mM)	18.8	0.627	0.146	0.826	5.59	8.0

**Fig. 5:**
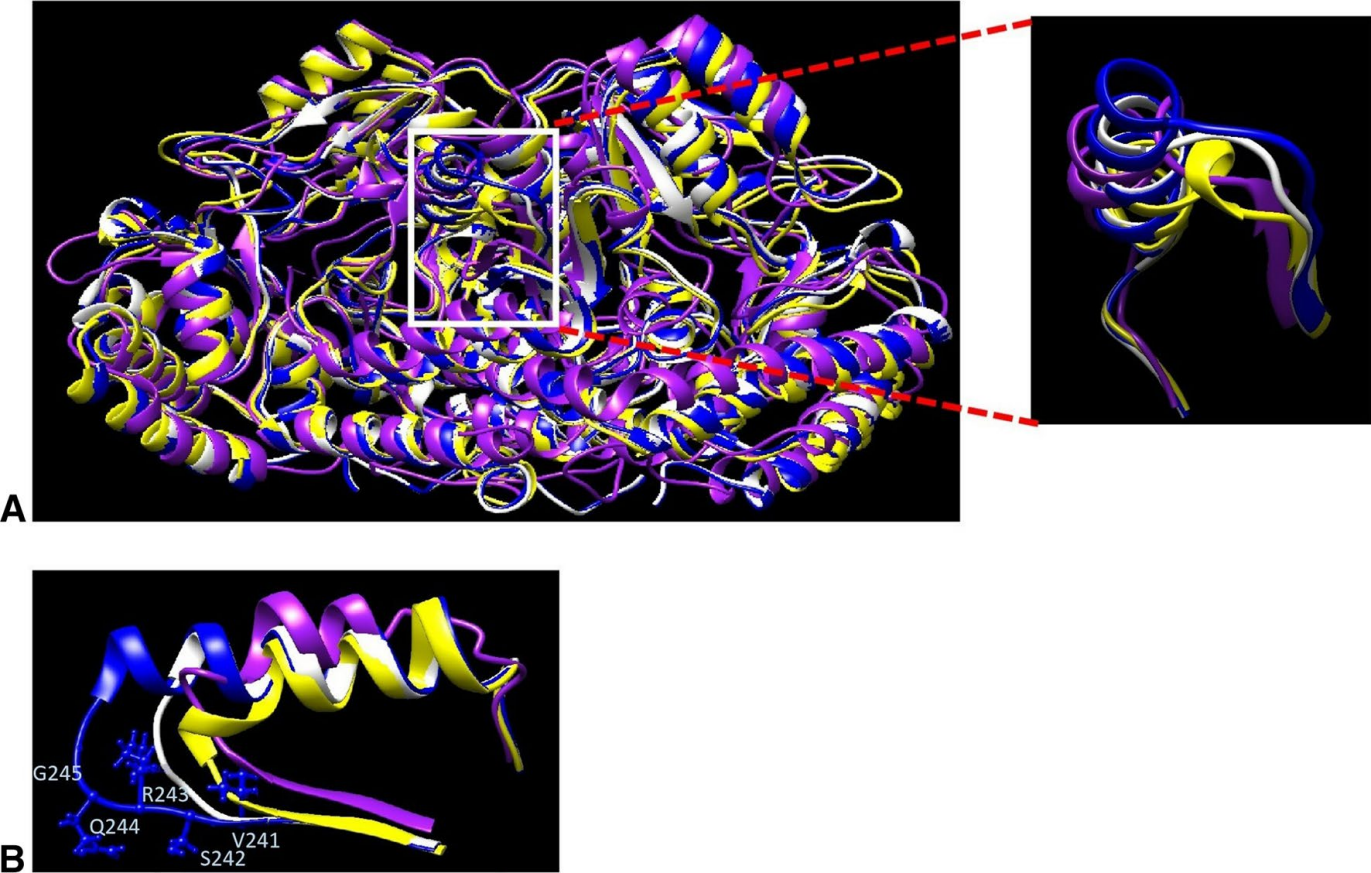
3D structure comparison of ITBSHMT_1 3D protein model (blue) and crystalized SHMTs (1dfo/SHMT from *E. coli* (white), 1kl2/SHMT from *B. stearothermophilus* (yellow) and 4uqv/SHMT from *M. janaschii* (purple)). **A**. Whole structure superimposition, **B**. Highlighted on ITBSHMT_1 additional fragment VSRQG on loop near PLP-binding site

Mutation on key binding residues is reported to have effect on SHMT’s structural stability and catalytic activity (Giardina et al. 2018). Based on secondary structure alignment with other characterized SHMTs, ITBSHMT_1 has conserve cofactors-binding residues (Fig S1). Mutation of one of the THF-binding sites (Y64A)  is predicted to produce decrease of PLG- and FFO-binding affinity but doesn’t affect structural stability. Mutation on one of the PLP-binding sites (Y65F)  is predicted to produce decrease of hydrogen bond but doesn’t affect PLG- and FFO-binding affinity. Double mutation (Y64A, Y65F) is predicted to produce decrease of hydrogen bond and FFO-binding affinity but increased PLG binding affinity (Table 2). Further structural analysis demonstrated that ITBSHMT_1 has novel structural feature (addition of 5 residues VSRQG on loop near PLG-binding site) (Fig S1). Superimposition of 3D structure protein model of ITBSHMT_1 and crystallized SHMTs (1dfo/SHMT from *E. coli*, 1kl2/SHMT from *B. stearothermophilus* and 4uqv/SHMT from *M. janaschii*) also revealed that this enzyme has bigger loop consisting of VSRQG residue (Fig. 5). Deletion of the fragment on ITBSHMT_1 is predicted to produce increase of hydrogen bond and salt bridge, decrease of hydrophobic cluster, produce significant decrease of PLG binding affinity but doesn’t affect FFO binding affinity. In conclusion, the unique fragment might have very essential function in maintaining of PLP binding with ITBSHMT_1. Some SHMTs also have been reported to have structural peculiarity. For example, unlike common SHMTs which have stoichiometry of 2 mol PLP/mol enzyme dimer, SHM1 from *M. tuberculosis* has stoichiometry of 1 mol PLP/mol enzyme dimer (Chaturvedi and Bhakuni 2003). Moreover, Ta0811 from *T. acidophilum* is defective in performing THF-dependent reaction because one of the folate binding loops is absent in the enzyme (Ma’ruf et al. 2021).

**Table 2:** Amounts of hydrogen bond, salt bridge and hydrophobic cluster on structure of 1dfo and 3D protein model of ITBSHMT_1 analyzed by ProteinTools server (https://proteintools.uni-bayreuth.de)

	Hydrogen bond	Salt bridge	Hydrophobic cluster	PLG affinity (kcal/mol)	FFO affinity (kcal/mol)
1dfo	87	27	22		
WT	173	59	25	− 7.3	− 10.3
Y64A	173	59	25	− 6.9	− 9.4
Y65F	169	59	25	− 7.3	− 10.3
Y64A, Y65F	170	59	25	− 7.7	− 8.9
Deletion VSRQG	183	61	24	− 5.9	− 10.3

The activity of some enzymes is influenced by the presence of metal ions and small organic compounds. It has been observed that SHMT activity is not significantly affected by alkaline metal (Na^+^, K^+^), alkaline earth metals (Mg^2+^, Ca^2+^), and chelating agent (EDTA), although it has been found to decrease rapidly in the presence of transition metals, especially Cu^2+^ (Jiang et al. 2013a; Jiang et al. 2013a; Yuan et al. 2014; Huang et al. 2015; Wang et al. 2022). This phenomenon has also occurred in ITBSHMT_1, thus suggesting that this enzyme does not use metal ion as a cofactor. To determine why transition metals, notably Cu^2+^, led to a significant decrease in enzyme activity, the enzyme’s Cu^2+^ binding sites were analyzed using MIB: Metal Ion-Binding site prediction and docking server (http://bioinfo.cmu.edu.tw/MIB/). The result revealed three Cu^2+^ binding sites in the catalytic pocket region (Table 3), some of which were part of Pyridoxal Phosphate Phenylserine (PLF)-binding residues, such as D200, H203 and H126 (Supplemental Fig S4). The binding of Cu^2+^ on these sites may prevent PLF from binding catalytic residues and hence inhibits enzyme activity.

**Table 3:** Cu^2+^ binding residues on ITBSHMT_1 (docking and analysis were performed using MIB server (http://bioinfo.cmu.edu.tw/MIB/)) and PLF binding residues analysis were performed using Ligplot plus software (Laskowski and Swindells, 2011))

No	Cu^2+^ binding sites
1	**D200**, **H203**
2	**H126**, H129, G173, S225
3	**H126**, H129, G173,

Similar binding residues between Cu^2+^ and PLF were indicated with bold words

Interestingly, the data on the effect of chemical reagents (Fig. 3C) also show that 30 mM of reducing agent led to an increase in ITBSHMT_1 activity. This occurred because β-mercaptoethanol may prevent oxydation of sulfhydryl group in the cysteine side chain to maintain enzyme activity. It has been reported that certain sulfhydryl reagents showed inactivated rabbit and *E. coli* SHMTs (Gavilanes et al. 1982, 1983; Schirch et al. 1985). Furthermore, a mutation of C410S on *E. coli* SHMT led to diminished binding of SHMTs substrate (Joshi-Tope and Schirch 1990). C410 plays a role in the structural stability of *E. coli* SHMT (C415 on ITBSHMT_1 corresponds to C410 on *E. coli* SHMT). In the presence of β-mercaptoethanol, could mean that C415 remains in reducing form (–SH) during the reaction process. Moreover, C384 and C415 may form a disulfide bridge (distance 11.96 Å) (Supplemental Fig S5A), which could affect the substrate- or cofactor-binding loop. As a comparison, the distance between C387 and C410 on the crystal structure of *E. coli* SHMT (PDB id: 1dfo) is longer (17.87 Å) than on ITBSHMT_1 (Supplemental Fig S5B). Therefore the presence of β-mercaptoethanol might also prevent disulfide formation on both *E. coli* SHMTs and ITBSHMT_1.

As a PLP-dependent enzyme, SMHT is a multifunctional enzyme that catalyzes the glycine and particular aldehyde reaction through an aldol condensation mechanism to produce β-hydroxy amino acid (Fig. 4A scheme 1) (Di Salvo et al 2013). The enzyme must have PLP as its main cofactor; depending on the reaction, there is also a partial need for THF as a second cofactor. For serine production, the enzyme usually requires THF as a cofactor (Jiang et al. 2013a; Jiang et al. 2013a; Yuan et al. 2014; Huang et al. 2015). Meanwhile, the synthesis of another β-hydroxy amino acid has been found to occur in the THF-independent reaction (Miyazaki et al. 1987; Kreuzman et al. 1997; Vidal et al. 2005; Gutierrez et al., 2008). The following findings comprise an exploration history of β-hydroxy amino acids synthesis using SHMTs. SHMT from *H. methylovorum* can catalyze the synthesis of hydroxy amino acid using aldehydes, such as acetaldehyde, benzaldehyde, p-hydroxy-benzaldehyde, and o-hydroxy-benzaldehyde at 25 °C (Miyazaki et al. 1987). *E. coli* SHMT catalyzes diastereo-specific condensation of glycine and various aldehydes to form the building blocks of carbachepam antibiotics. At a high temperature (61 °C), *E. coli* SHMT catalyzes condensation between glycine and various non-natural aldehydes, such as succinic semi-aldehyde methyl ester (SSAME), succinic semi-aldehyde ethyl ester (SSAEE), and succinic semi-aldehyde tri-butyl ester (SSATBE). In contrast, at a low temperature (15 °C), the enzyme catalyzes condensation between glycine and pentenal (Kreuzman et al. 1997). SHMT from *Streptococcus thermophilus* has also been used to catalyze β-hydroxy amino acid formation produced from the condensation of glycine and non-natural aldehydes, such as benzyloxyacetaldehyde and N-Cbz-alanine at 37 °C (Vidal et al. 2005). Further characterization of *S. thermophilus* SHMT has shown that the enzyme can condense glycine and various aldehydes, such as N-(R)-, N-(S)-Cbz-alanine, NCbz-2-aminoethanol, N-Cbz-3-aminopropanal, and benzyloxyacetaldehyde, at 4 °C (Gutierrez et al., 2008). Recently, SHMT from *Pseudomonas plecogossicida*, *Shewanella algae*, *Alcanivorax* sp., and *Arthrobacter* sp. has been used to catalyze the serine synthesis through the condensation cycle of THF and formaldehyde at 34, 34, 35, and 30 °C respectively (Jiang et al. 2013a; Jiang et al. 2013a; Yuan et al. 2014; Huang et al. 2015). In serine synthesis, the ability of ITBSHMT_1 to use the condensation cycle of THF and formaldehyde (Fig. 4A scheme 2) and threonine and phenylserine synthesis in THF-independent reactions (Fig. 4A schemes 3 and 4) was monitored. In line with previous analysis, the addition of β-mercaptoethanol elevated serine, threonine, and phenylserine production (Table 4). Moreover, in line with phenylserine cleavage (Fig. 3B), the optimum temperature for the synthesis reaction of those β-hydroxy amino acids was also 80 °C (Fig. 4B). This result might give new findings in the field of thermostable multipurpose biocatalyst since there is only limited exploration of thermostable SHMT for the synthesis of these β-hydroxy amino acids.

**Table 4:** Influence of β-mercaptoethanol/BME and Tetrahydrofolate/THF on specific activity of serine, threonine and phenylserine synthesis (80 °C and pH 7.5)

	Specific Activity (µmol /min.mg)		
	Serine	Threonine	Phenylserine
– BME – THF	N/D	1.1 ± 0.14	0.62 ± 0.04
+ BME – THF	N/D	4.09 ± 0.28	2.82 ± 0.1
– BME + THF	3.01 ± 0.11	0.62 ± 0.07	0.43 ± 0.02
+ BME + THF	45.99 ± 0.65	4.79 ± 0.12	3.04 ± 0.11

All experiment is considered as significant (*P* value ≤ 0.05) except for comparison between phenylserine synthesis + BME –THF and + BME + THF. (–): without addition, (+): with addition

## Conclusion

ITBSHMT_1 from *P. taiwanensis* AL17 was successfully expressed in an active form using *E. coli* Rosetta 2 (DE3) as the host cell and purified using the NiNTA affinity chromatography system. Homology analysis revealed that like common SHMT, ITBSHMT_1 contains PLP- and THF-binding residues. Bio-computational and biochemical analysis revealed that this enzyme is catalytically active as a dimer protein. It exhibited optimal activity at pH 7.5 and 80 °C and has a higher catalytic efficiency than SHMTs from mesophilic and psychrophilic bacteria. Additional analysis demonstrated that ITBSHMT_1 does not a metallo-enzyme since its activity is not enhanced by the presence of metal ions, and EDTA does not affect its activity. In contrast, transition metal ions, especially Cu^2+^, led to a significant decrease in enzyme activity. Biocomputational analysis revealed that Cu^2+^ will bind to PLF binding residues, preventing the substrate from binding on the enzyme. The addition of β-mercaptoethanol to the enzymatic reaction revealed that the presence of the reducing agent will affect the structure of ITBSHMT_1 and thus enhance its activity. Structural analysis reveals that ITBSHMT 1 has 5 additional residues VSRQG on a loop near the PLP-binding site that might have very essential role in PLP binding affinity. ITBSHMT_1 demonstrated catalytic versatility in producing various β-hydroxy amino acids, such as serine, threonine, and phenylserine, with optimum activity at 80 °C. Biocomputational analysis revealed that the higher number of hydrogen bonds, salt bridges, and hydrophobic clusters in the 3D structure of ITBSHMT_1 compared to mesophilic SHMT from *E. coli* (1dfo) might be correlated with the enzyme’s high thermal stability.

## Supplementary Information

Below is the link to the electronic supplementary material.

Supplementary file1 (DOCX 1749 KB)

## Data Availability

Nucleotide sequence of ITBSHMT_1 has been deposited in National Center for Biotechnology Information (https://www.ncbi.nlm.nih.gov/) with accession number MN688201.1.
