# Supplementary material for: Characterization of thermostable serine hydroxymethyltransferase for β-hydroxy amino acids synthesis
Source: Amino Acids. 2022 Dec 17;55(1):75–88. doi: 10.1007/s00726-022-03205-w (PMC9876860; doi:10.1007/s00726-022-03205-w)
Supplement: Supplementary file 1 — Supplementary file1 (DOCX 1749 KB) [file 726_2022_3205_MOESM1_ESM.docx]

**SUPPLEMENTARY MATERIAL**

**Submit to Amino Acids**

**Characterization of Thermostable Serine Hydroxymethyltransferase for β-hydroxy Amino Acids Synthesis**

Ilma Fauziah Ma’ruf.^1^, Elvi Restiawaty^2^, Syifa Fakhomah Syihab^1,3^, Kohsuke Honda^4^, and Akhmaloka^1,5*^

1. Doctoral Program of Chemistry, Faculty of Mathematics and Natural Sciences, Institut Teknologi Bandung, Indonesia
2. Chemical Engineering Process Design and Development Research Group, Faculty of Industrial Technology, Institut Teknologi Bandung, Indonesia
3. Faculty of Sports and Health Education, Universitas Pendidikan Indonesia, Indonesia
4. International Center for Biotechnology, Osaka University, Japan
5. Department of Chemistry, Faculty of Science and Computer, Universitas Pertamina, Jakarta, Indonesia

^*^Corresponding author:

Akhmaloka

Biochemistry Research Group

Faculty of Mathematics and Natural Science

Institut Teknologi Bandung

Phone :+62 22 2502103

Fax :+62 22 2504154

Email : loka@chem.itb.ac.id

**Suppelemental Figure Legends**

**Figure S1.** Secondary structure alignment of ITBSHMT_1 and characterized SHMTs generated by using Promals3D website (http://prodata.swmed.edu/promals3d/). Conservation score: 5-9. Consensus predicted secondary structure symbols: alpha-helix: h; beta-strand: e. Consensus amino acid symbols are: conserved amino acids are in **bold and uppercase** letters; aliphatic (I, V, L): *l*; aromatic (Y, H, W, F): *@*; hydrophobic (W, F, Y, M, L, I, V, A, C, T, H): *h*; alcohol (S, T): o; polar residues (D, E, H, K, N, Q, R, S, T): p; tiny (A, G, C, S): t; small (A, G, C, S, V, N, D, T, P): s; bulky residues (E, F, I, K, L, M, Q, R, W, Y): b; positively charged (K, R, H): **+**; negatively charged (D, E): **-**; charged (D, E, K, R, H): c. yellow: PLP binding residues, brown: THF binding residues, green: unique fragment VSRQG in ITBSHMT_1.

**Figure S2.** 3D structure model of ITBSHMT_1 built using SWISS-MODEL server (Waterhouse et al, 2018). SHMT monomer (red and blue), PLP and THF binding sites (white circle) and cysteine residues (yellow) were indicated.

**Figure S3.** Ligand-protein interaction generated using Autodock Vina software (Eberhardt et al, 2021) and visualized using Ligplot plus software (Laskowski and Swindells, 2011). A. ITBSHMT_1-PLG, B.ITBSHMT_1-FFO, C.ITBSHMT_1-TGF. Ligand (blue stick), ligand binding residues forming hydrogen bond (black circle), hydrogen bond (green dash, distance in Angstrom (Å)), hydrophobic interaction (red tassel), carbon atom (black ball), nitrogen atom (blue ball) and oxygen atom (red ball) were indicated.

**Figure S4.** ITBSHMT_1-PLF and Cu^2+^ interaction generated using Autodock Vina software (Eberhardt et al, 2021) and visualized using Ligplot plus software (Laskowski and Swindells, 2011). Ligand (blue stick), Cu^2+(^green ball) ligand binding residues forming hydrogen bond (black circle), hydrogen bond (green dash, distance in Angstrom (Å)), hydrophobic interaction (red tassel), carbon atom (black ball), nitrogen atom (blue ball) and oxygen atom (red ball) were indicated.

**Figure S5.** Orientation of cysteine risidues, visualized using UCSF Chimera software (Petterson et al, 2004), distance measured in Angstrom (Å)

**A.** Distance between C384 and C415 on ITBSHMT_1

**B.** Distance between C381 and C410 on 1dfo

**Figure S1**

**
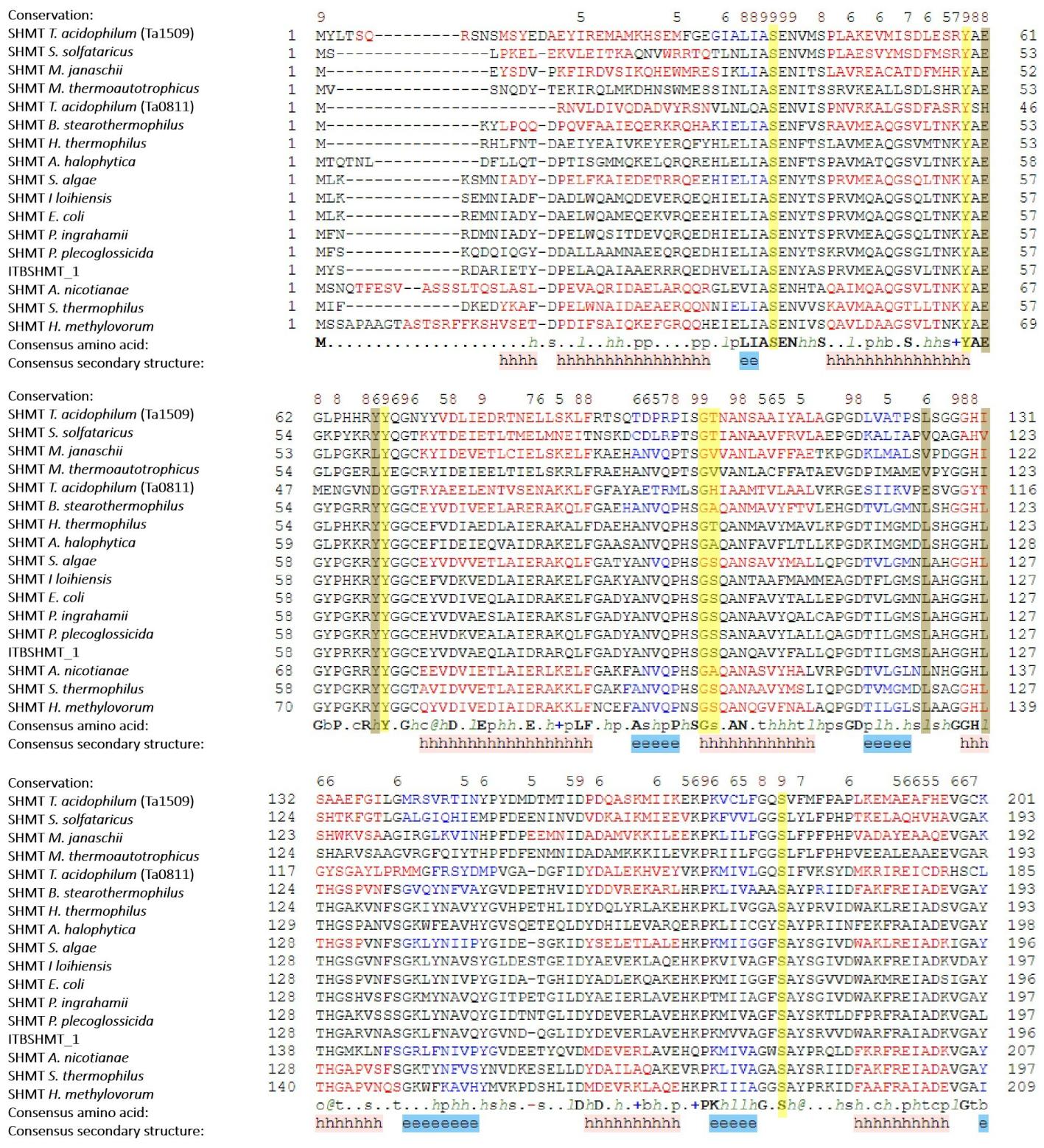
**

**
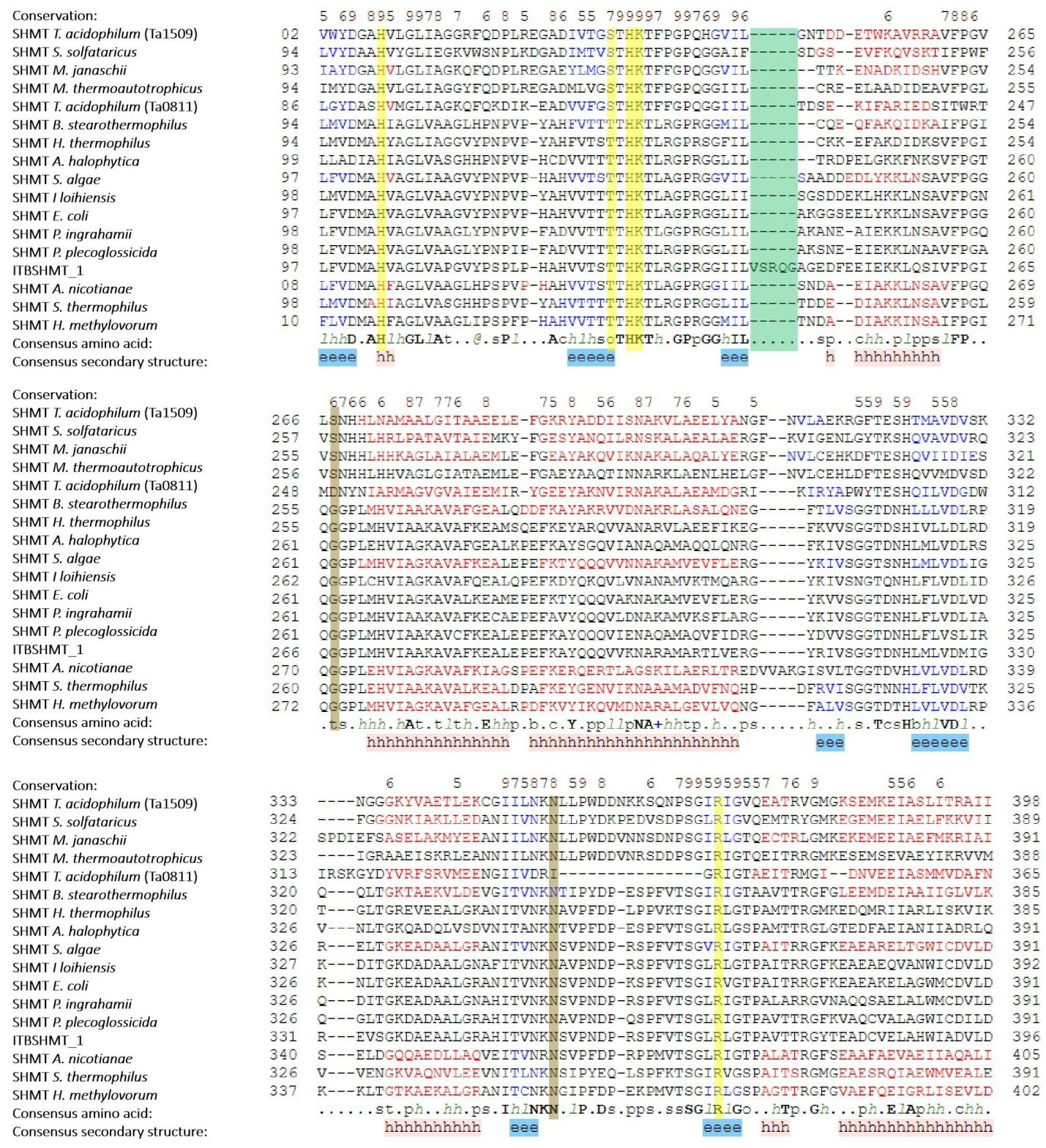
**

**
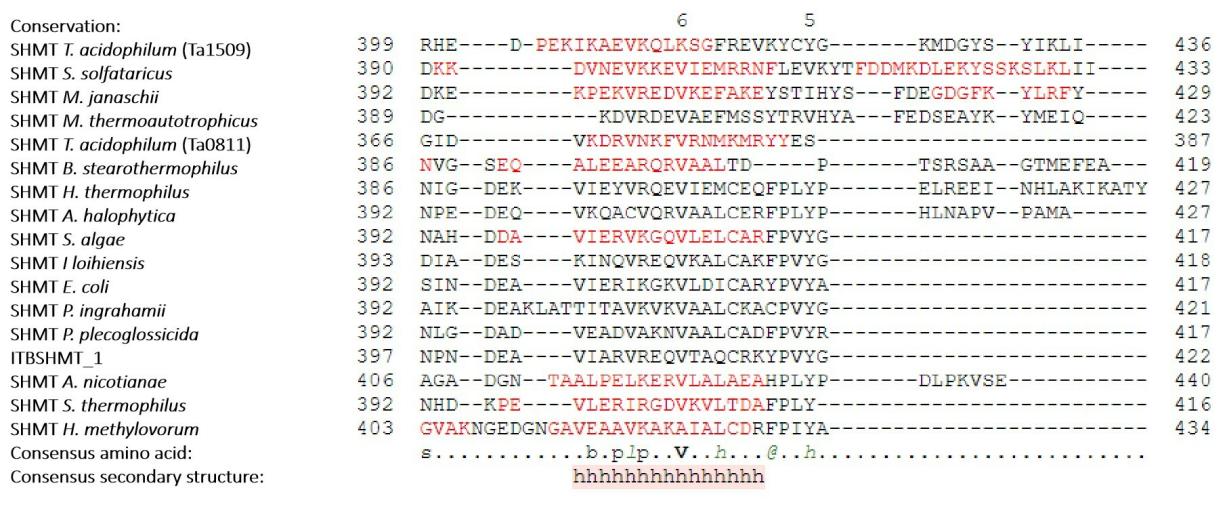
**

**Figure S2**

**
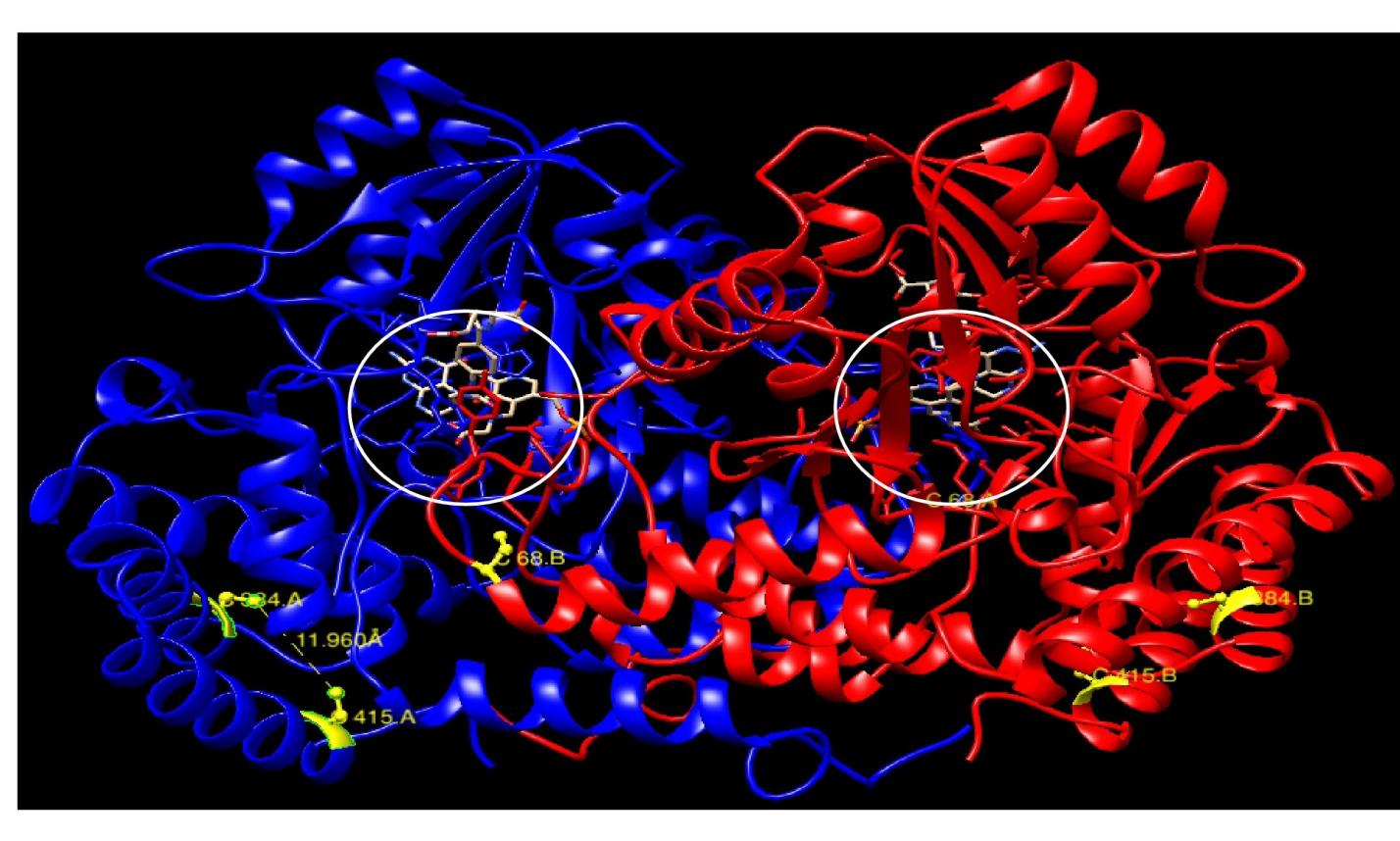
**

**Figure S3**

**
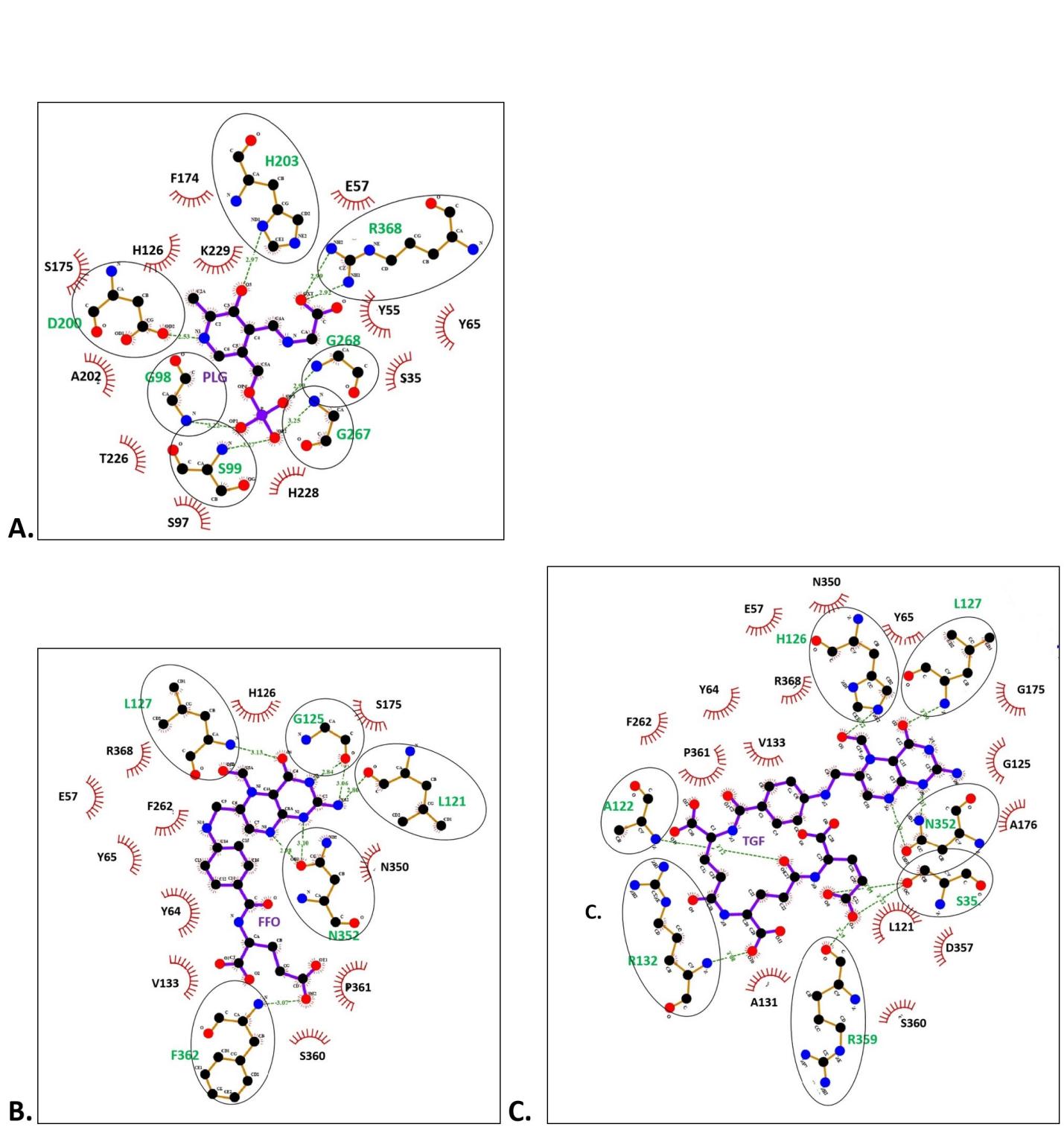
**

**Figure S4**

**
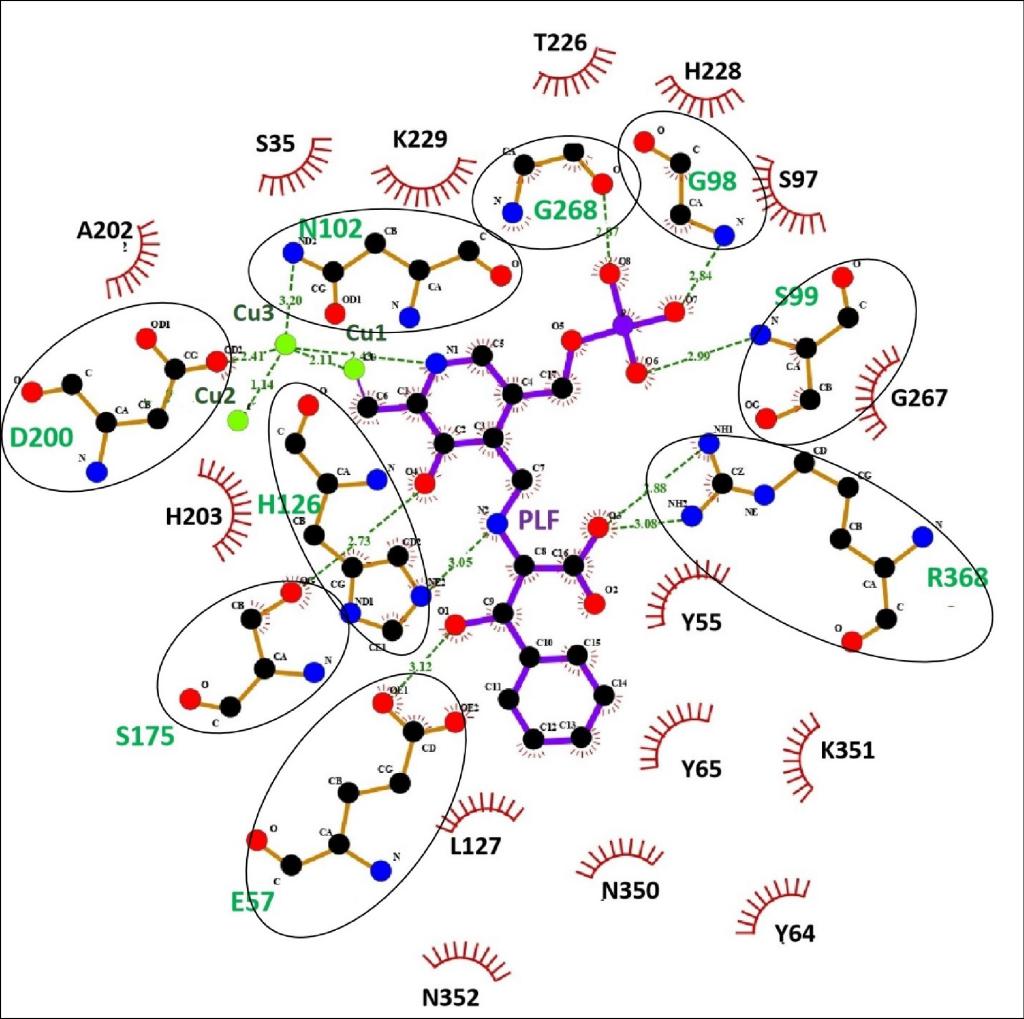
**

**Figure S5**

**
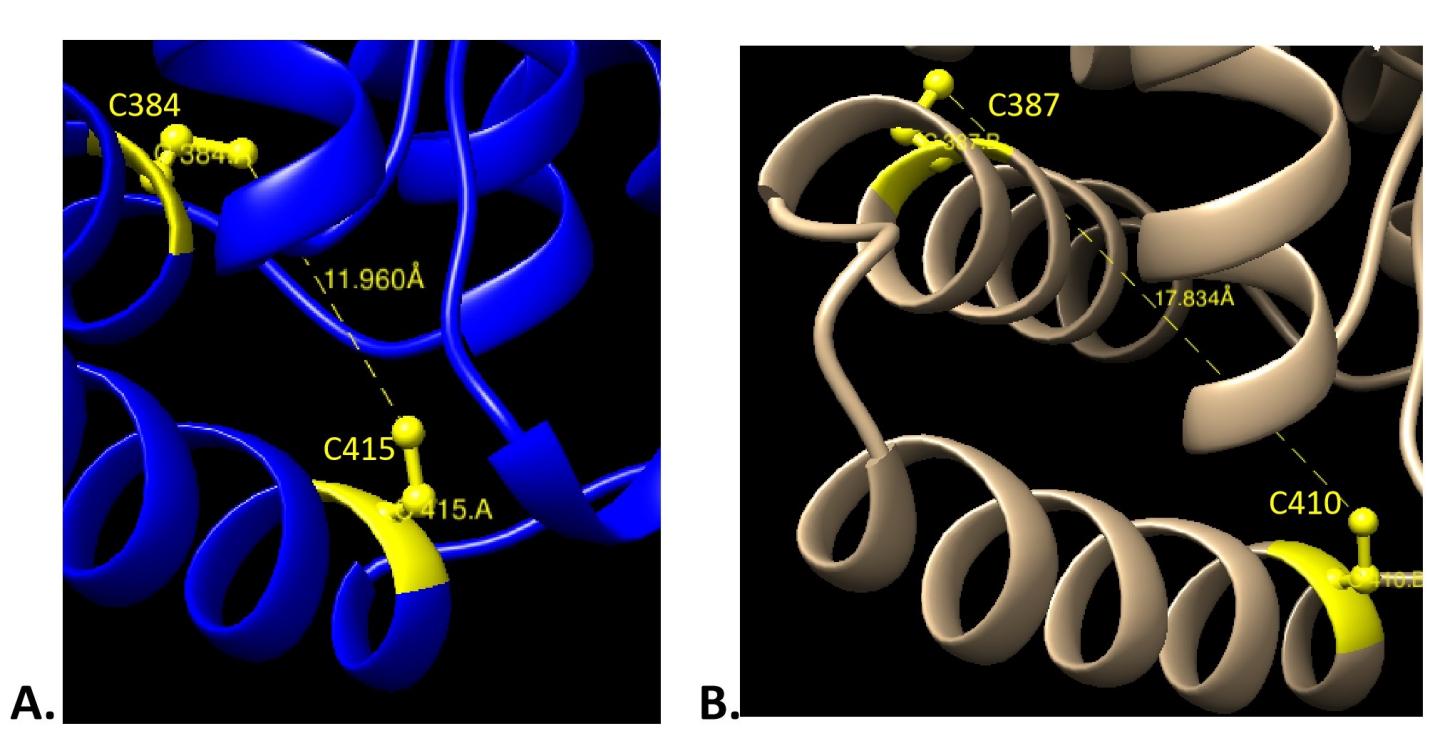
**

**Table S1.** Activity of ITBSHMT_1, CE: crude extract, P: purified (70 ˚C and pH 7.5) .

|  | **Volume (mL)** | **Total  activity (U)** | **Total  protein (mg)** | **Specific  activity (U/mg)** | **Yield (%)** | **Purification fold** |
| --- | --- | --- | --- | --- | --- | --- |
| **CE** | 6,2 | 209,916 | 5,263 | 39,884 | 100 | 1 |
| **P** | 5,5 | 186,312 | 1,212 | 153,620 | 88,755 | 3,851 |

**Table S2. PLP and THF binding residues on ITBSHMT_1 subunit A (A) and B (B)**

| **PLP binding residues on subunit A** | S35, Y55, Y65 (B), Y70 (B), G98, S99, H126, S175, D200, H203,T226, H228, K229 G268(B) and R368 |
| --- | --- |
| **THF binding residues on subunit A** | E57, Y64 (B), L121, G125, L127 and N353 |
| **PLP binding residues on subunit B** | S35, Y55, Y65 (A), Y70 (A), G98, S99, H126, S175, D200, H203,T226, H228, K229 G268(A) and R368 |
| **THF binding residues on subunit B** | E57, Y64 (A), L121, G125, L127 and N353 |
